# Application of Metagenomics Sequencing in a Patient with Dementia: A New Case Report

**DOI:** 10.3390/genes15081089

**Published:** 2024-08-18

**Authors:** Maria Minelli, Federico Anaclerio, Dario Calisi, Marco Onofrj, Ivana Antonucci, Valentina Gatta, Liborio Stuppia

**Affiliations:** 1 Center for Advanced Studies and Technology (CAST), “G. d’Annunzio” University of Chieti-Pescara, 66100 Chieti, Italy; mariaminelli60@gmail.com (M.M.); dariocalisi95@outlook.it (D.C.); i.antonucci@unich.it (I.A.); v.gatta@unich.it (V.G.); stuppia@unich.it (L.S.); 2 Department of Medical Genetics, “G. d’Annunzio” University of Chieti-Pescara, 66100 Chieti, Italy; 3 Department of Neurosciences, Imaging and Clinical Sciences, “G. d’Annunzio” University of Chieti-Pescara, 66100 Chieti, Italy; onofrj@unich.it; 4 Department of Psychological, Health and Territorial Sciences, “G. d’Annunzio” University of Chieti-Pescara, 66100 Chieti, Italy

**Keywords:** microbiome, metagenomics, NGS, dementia, neurodegenerative disease, 16S

## Abstract

(1) Background: The study of the microbiome is crucial for its role in major systemic diseases, in particular the oral and gut microbiota. In recent years, the study of microorganisms correlated, for example, with neurodegenerative disease has increased the prospect of a possible link between gut microbiota and the brain. Here, we report a new case concerning a patient who was initially evaluated genetically for dementia and late-onset dyskinesia, and later tested with 16S metagenomics sequencing. (2) Methods: Starting from a buccal swab, we extracted bacterial DNA and then we performed NGS metagenomics sequencing based on the amplification of the hypervariable regions of the 16S rRNA gene in bacteria. (3) Results: The sequencing revealed the presence of the *Spirochaetes* phylum, a pathogenic bacterium generally known to be capable of migrating to the Central Nervous System. (4) Conclusions: Oral infections, as our results suggest, could be possible contributing factors to various neurodegenerative conditions.

## 1. Introduction

**Figure 1:**
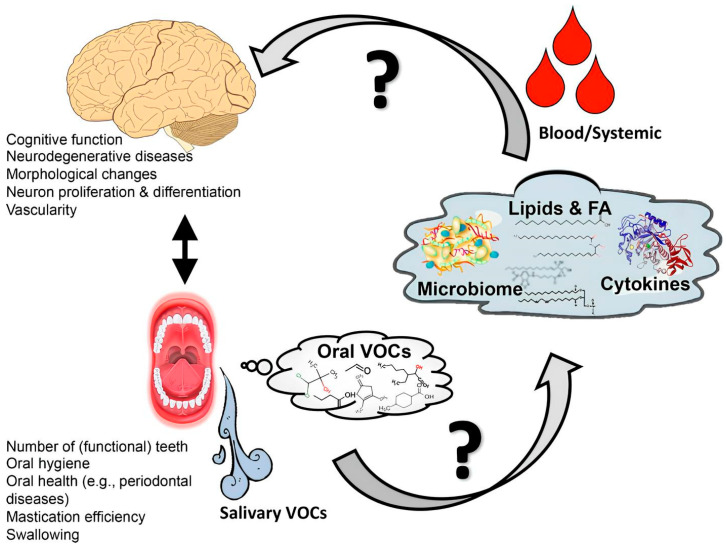
The possible mechanism and interaction between oral microbiota, brain, and health function.

The human microbiome is known to play a role in the development of major systemic diseases. In particular, experimental and clinical studies suggest a possible link between these biomarkers, oral health, and cognitive decline. The mechanism by which the oral microbiota, the second most diverse and populous in the mammalian body (as shown in Figure 1 by Orr, Miranda E et al., 2020), affects cognition is likely mediated by modifications in the oral microenvironment that select for pathogens and facilitate the transmission of bacteria outside the mouth [1].

Different species of *Spirochaetes* have been correlated with various neurodegenerative diseases and other pathologies, particularly the genus *Treponema*, a microaerophilic bacterium that survives for a short time outside the infected organism [2,3]. It is closely related (>99% DNA homology) to other species within the *Spirochaetes* phylum. Pathogenic *Treponemes* are not culturable in the laboratory, unlike nonpathogenic *Treponemes*; in fact, we can distinguish: *T. pallidum*, the etiologic agent of syphilis [4]; *T. carateum* which causes pinta [5]; *T. pertenue*, the etiologic agent of framboesia [6]; and *T. endemicum* that causes bejel or “endemic syphilis” [7]. Previous reports have also documented the amplification of *T. pallidum* nucleic acid from oral or pharyngeal swabs [8,9], or in one instance, saliva [10], in individuals with syphilis.

**Figure 2:**
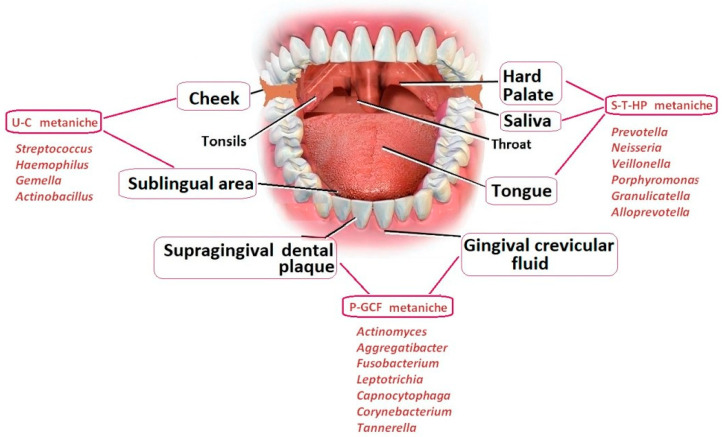
The different areas and the typical bacteria that could potentially invade the oral microbiota.

Actually, *Spirochaetes* are highly neurotropic and can spread along nerve fibers and the lymphatic system. They have been identified in the trigeminal nerve and ganglia, which might be the preferential access to the brain. It has also been shown that typical oral species of the phylum *Spirochaetes* (including multiple species of the genus *Treponema*) often comprise amyloid plaques (as shown in Figure 2 by Bacali C et al., 2022) [11].

In 2021, Su et al. confirmed that oral *T. denticola* can induce β-amyloid accumulation in the hippocampus of C57BL/6 mice [12].

In this study, we present a case of a patient with dementia who was tested for Huntington’s disease, which resulted in a normal number of CAG repeats in the HTT gene. However, oral microbiome analysis from buccal swabs revealed the presence of a novel phylum, the *Spirochaetes* phylum, which we had not previously encountered, with 20% of the microbiome consisting of the species *T. denticola*. We hypothesize that, in addition to genetic factors, there may be a specific condition associated with dementia in this patient that contributes to a pro-inflammatory aging process (proinflammaging). Our objective is to investigate whether the presence of this microbial phylum in the oral microbiome could be linked to such a condition, potentially offering new insight into the role of the microbiome in neurodegenerative disease.

## 2. Case Presentation

We describe the case of an 83-year-old woman who was evaluated genetically due to dementia and late-onset dyskinesia. Initially, the neurologist made the diagnosis of Lewy Body Dementia but then she started to present akathisia, and somatic dyskinesia involving even the diaphragm and the abdomen. Moreover, she suffered from a sleep disorder, complex mood disorder, psychosis including unintelligible confabulations, and Parkinsonism. Her most recent head CT scan revealed signs of marked cerebral atrophy and slight pallidal calcifications. Her medical history included previous deep venous thrombosis, pulmonary embolism, and a femoral fracture. She is being treated with dabigatran etexilate, tetrabenazine, levodopa benserazide, astazin, quietapine, and lorazepam. She was an elementary school teacher and there was no clear family history of neurodegenerative disorders. She is a widow and has two sons. Genetic testing for Huntington’s disease revealed a normal number of CAG repeats. However, when we processed the extraction of DNA to perform the analysis, we noticed an unparalleled reaction in the filter column that we usually use for the buccal swab with the buffer reaction. Generally, when we perform the extraction of the DNA, we obtain a transparent color of the elution of DNA. In this case, after centrifugation and before proceeding with the automatic extraction of DNA, we observed a black volume of elution. This led us to consider whether any medications the patient was taking might have interfered with the reaction. After a careful literature review, we came to no conclusion and consequently decided to proceed with an analysis of the oral microbiota to assess the presence of possible pathogens.

## 3. Materials and Methods

Starting from the buccal swab, bacterial DNA was extracted using the MagPurix instrument and the Bacterial DNA Extraction Kit (Zinexts Life Science Corp., Taipei, Taiwan-CatZP02006) according to the manufacturer’s protocol. To ensure the accuracy and reliability of the metagenomic sequencing results, we included both negative and positive controls in our analysis. Negative controls, such as blank swabs and extraction blanks, were used to detect any potential contamination introduced during the sample processing and sequencing stages. Positive controls, consisting of known microbial communities, were included to verify the efficacy and sensitivity of the sequencing process. These controls allowed us to validate the presence of the detected microbial taxa and ensure that the results were not artifacts of contamination. The bacterial DNA was quantified with a Qubit 3.0 fluorometer (ThermoFisher, Waltham, MA, USA). NGS was performed with the Ion 16S™ Metagenomics Kit (ThermoFisher, Waltham, MA, USA), designed for the analyses of mixed microbial populations using the Ion Torrent™ semiconductor sequencing workflow. The kit permits PCR amplification of the hypervariable regions of the 16S rDNA gene from bacteria. The kit includes two primer sets that selectively amplify the corresponding hypervariable regions of the 16S region in bacteria:

- Primer set V2-4-8;
- Primer set V3-6, 7-9.

After the quantification of libraries with the Real-Time Step One PCR System (Thermo Fisher Scientific, Waltham, MA, USA), the amplified fragments can then be sequenced using the Ion S5™ (ThermoFisher, Waltham, MA, USA) platform, loaded onto an Ion 520TM chip and analyzed using the Ion 16S™ metagenomics analyses module within the Ion Reporter™ software 5.14 (ThermoFisher, Waltham, MA, USA), enabling a rapid and semi-quantitative assessment of complex microbial samples. These comprehensive primer sets allow for accurate detection and identification of a broad range of bacteria down to the genus or species level. The primer sets are paired with Environmental Master Mix v2.0 that is optimized to tolerate high levels of PCR inhibitors and amplify targets from complex samples such as environmental, food, tissue, and other challenging samples. The combination of the two primer pools allows for the sequence-based identification of a broad range of bacteria within a mixed population.

## 4. Results

**Figure 3:**
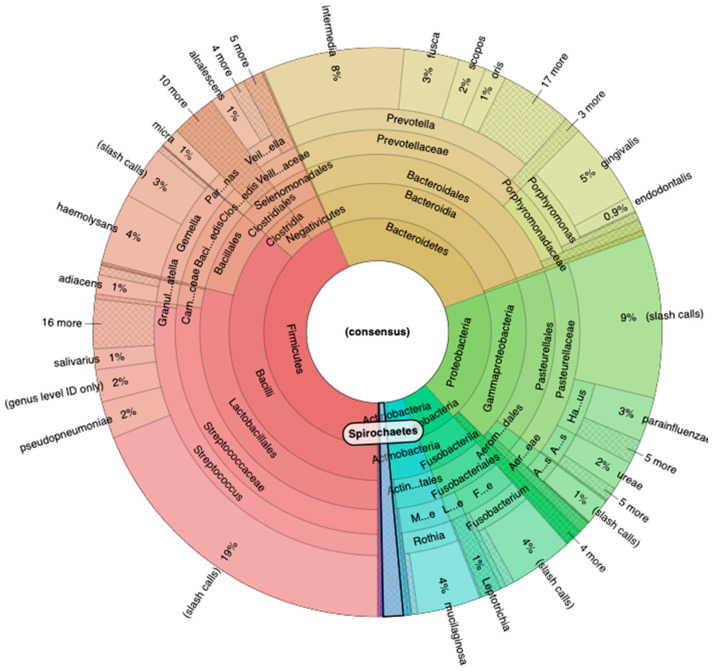
The result of 16S Metagenomics sequencing that revealed the presence of *Spirochaetes* phylum.

The sequencing performed with the 16S Metagenomics Kit revealed an important result. Using the Ion Reporter Software with the Krona software, we analyzed all the phylogenetic classes, starting from the major phyla. In particular, the phylum *Firmicutes* revealed 43% of the entire microbial population, while the phylum *Bacteroidetes* 26%, the phylum *Proteobacteria* 19%, the phylum *Fusobacteria* 6%, and *Actinobacteria* 4% (Figure 3).

**Figure 4:**
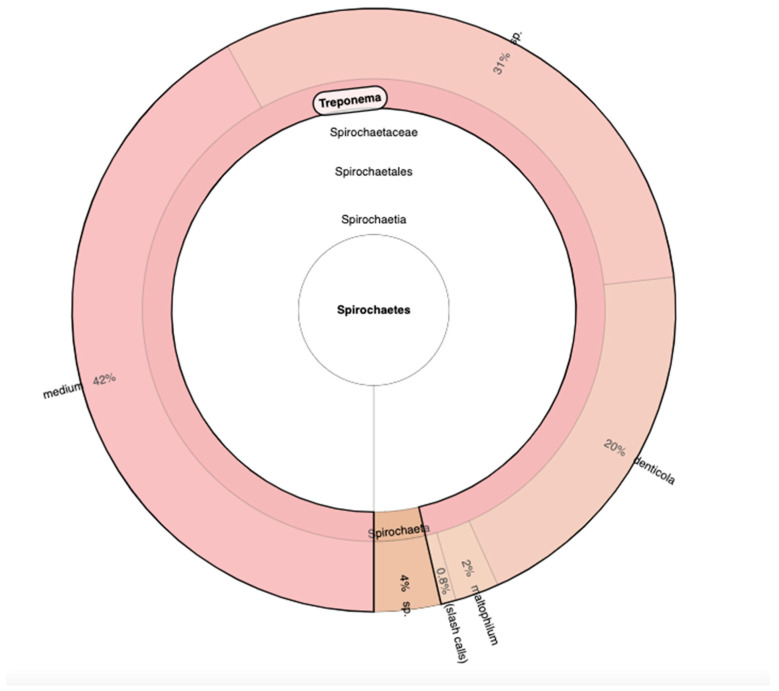
The analysis of Spirochaetes phylum with the Krona software to comprehend the different genus and species.

Due to the characterization of all the bacteria communities based on hypervariable regions, we discovered and detected with primer V2, V3, V6, V7, and V8 the presence of a new phylum that we had never encountered, the *Spirochaetes* phylum. In particular, the genus *Treponema* was over 90% of the entire phylum. Continuing the phylogenetic analysis, the most abundant species are *T. medium* with 42%, *Treponema* sp. with 31%, and *T. denticola* with 20% (Figure 4).

## 5. Discussion

Different microorganisms belonging to the oral and intestinal microbiota have been linked to various diseases. Within the *Spirochaetes* phylum, *T. denticola* is known to be able to migrate to the Central Nervous System (CNS) along the peripheral and central nerves, like the trigeminal pathway, which bypasses the brain–blood barrier control, or through lymphatic vessels, as evidenced by the presence of the spirochetal chemokine CXCL13 in high concentrations in the cerebrospinal fluid but not in the serum. It is the larger bacterium upon which other pathogens, like *P. gingivalis*, could easily enter the CNS due to the marked neurotrophism displayed by all *Spirochaetes*. The above-mentioned pathway is likely to evade the initial immune recognition of these microorganisms because of their ability to hide within the ganglia, perhaps for many years, before invading the neighboring areas of the brain, such as the locus coeruleus, thereby affecting neurotransmitter release in the host [13]. Recent research suggests that oral infections could also be possible contributing factors to various neurodegenerative conditions [14].

More studies are required to establish a causal link between oral infections and neurodegeneration. Current research relies on relatively small samples and cross-sectional surveys, which only allow for hypotheses regarding the presence of an oral–brain connection. Therefore, most findings still seem preliminary, as their authors generally acknowledge. To elucidate the nature of the connection between the oral microbiome and the CNS, it is crucial to create and validate a methodological approach grounded in a precise definition of oral dysbiosis and the mental disorders analyzed. The identification of specific oral dysbiosis signatures associated with various mental disorders raises the question of whether the CNS modulates the oral microbiome. This would constitute the complementary element of a bidirectional relationship, suggesting the existence of an oral–cerebral axis equivalent to the gastrointestinal axis. If the bidirectional connection between the oral microbiome and the CNS were confirmed, rebalancing microbial homeostasis with probiotics could offer a potential therapeutic pathway [15]. Although our study cannot yet definitively establish the causal role of our findings, it suggests some associations between oral microbiota and cognitive disorders or dementia. The identification of the *Spirochaetes* phylum in the patient’s oral microbiome is of particular interest due to its potential role in neuroinflammatory processes. Previous studies have suggested that certain species within this phylum, including *T. denticola*, may be involved in the pathogenesis of neurodegenerative diseases through mechanisms such as chronic inflammation and the disruption of the blood–brain barrier. While this case report cannot establish a direct causal relationship, the presence of *Spirochaetes* raises the possibility that oral pathogens might contribute to the progression of dementia by promoting neuroinflammation.

The patient’s microbial profile might serve as a biomarker for evaluating disease risk, early treatment, assessing therapy response, or guiding new treatments and preventive measures [11].

From a clinical perspective, these findings suggest that oral health and microbiome composition should be considered as potential factors in the management of patients with neurodegenerative diseases. Further research is needed to determine whether targeting oral pathogens could become a viable strategy in the prevention or treatment of conditions such as dementia. Additionally, routine screening for specific microbial signatures in the oral cavity could eventually serve as a non-invasive diagnostic tool to identify patients at higher risk of developing neuroinflammatory disorders.

## 6. Conclusions

Future microbiome analysis could become a novel approach for preventing systemic diseases and for applying possible therapies in individuals with dysbiosis or with the presence of pathogenic microorganisms, especially in the era of precision medicine. One limitation of our study is the potential impact of the patient’s medications on the oral microbiota composition. Certain medications, especially those commonly prescribed for neurodegenerative diseases, can alter the microbial community in the oral cavity, potentially influencing our results. Future studies should investigate the role of medication in shaping the oral microbiome. Another significant limitation is the use of a single case report, which limits the generalizability of our findings. While the discovery of the *Spirochaetes* phylum in this patient is intriguing, it cannot be conclusively linked to dementia or neuroinflammation based on one case alone. Larger studies are needed to validate these findings and explore their relevance in a broader population. This field is still underexplored, and further research with larger sample sizes and involving various types of mental diseases is necessary to clarify the biological connection or interactions between the oral microbiota and all mental health disorders, and to develop consistent patterns to generate more definitive data.

Our study highlights the need for further research to understand the role of the oral microbiome in neurodegenerative diseases. Future investigations should include larger sample sizes, longitudinal studies, and detailed analyses of other potential influencing factors, such as diet, genetics, and environmental exposures.

## Data Availability

Data available upon request.
